# Effects of Probiotics Supplementation on Gastrointestinal Symptoms in Athletes: A Systematic Review of Randomized Controlled Trials

**DOI:** 10.3390/nu14132645

**Published:** 2022-06-26

**Authors:** Karolina Łagowska, Joanna Bajerska, Szymon Kamiński, Cristian Del Bo’

**Affiliations:** 1 Department of Human Nutrition and Dietetics, Poznań University of Life Sciences, Wojska Polskiego 31, 60-624 Poznan, Poland; joanna.bajerska@up.poznan.pl (J.B.); s_z_k@wp.pl (S.K.); 2 Department of Food, Environmental and Nutritional Sciences (DeFENS), Università degli Studi di Milano, 20133 Milan, Italy; cristian.delbo@unimi.it

**Keywords:** gastrointestinal symptoms, endurance athletes, probiotic supplementation

## Abstract

This study examines the effectiveness of probiotic supplementation on gastrointestinal (GI) symptoms, the gut barrier function, and inflammatory markers in athletes based on data from randomised controlled trials. Searches were conducted in PubMed, the Cochrane Library, and the Web of Science up to October 2021. The protocol for this review was registered with PROSPERO (CRD42021284938). Two reviewers independently screened the titles, abstracts, and full texts to identify articles on the influence of probiotics or symbiotics on GI symptoms, gut barrier function, and cytokines, and the quality of the studies was assessed using RoB2. Ten articles involving 822 athletes were included in this review. A single strain *Lactobacillus* bacteria was used in three studies, seven studies used a *Lactobacillus* and *Bifidobacterium* multi-strain cocktail, and one study used this cocktail with a prebiotic. Only slight evidence was found for a positive effect of probiotics on GI symptoms in athletes during training, exercise, and competition, so it was not possible to identify the best product for managing GI symptoms in athletes. Due to the small number of studies, it was also difficult to find a direct association between the reduced exercise-induced perturbations in cytokines, gut barrier function, and GI symptoms after probiotic supplementation.

## 1. Introduction

Gastrointestinal (GI) symptoms are widely reported among athletes participating in prolonged endurance training and competition, and they may significantly decrease physical performance and affect results. Pugh et al. showed that 27% of marathon runners have moderate to more severe GI symptoms while competing [1]. Evidence has shown that even moderate-intensity exercise of short duration can compromise the gastrointestinal tract and promote the occurrence of gastrointestinal symptoms [2].

A variety of complaints may occur during exercise, which are attributed to disorders of the upper GI tract (the oesophagus and stomach), as well as the lower part of the GI tract (the small intestine and the colon) [3]. Complaints of the upper GI include reflux, nausea, bloating, and upper abdominal cramping, whereas the lower GI complaints include lower abdominal cramping, the urge to defecate, and increased frequency of bowel movements, flatulence, and diarrhoea [4,5,6]. The aetiology of these disturbances has not been fully elucidated, and while it is recognised to be multifactorial, GI ischaemia is often acknowledged as the main pathophysiological mechanism for such symptoms [3,5].

Some authors believe that these problems are associated with alterations in intestinal permeability and decreased gut barrier function. Increased GI permeability, sometimes referred to as a ‘leaky gut’, also leads to endotoxemia, increased inflammatory status, and a higher level of cytokines [7]. It should also be mentioned that strenuous exercise leads to a robust inflammatory response, mainly characterised by an increase in circulating inflammatory mediators which are produced by immune cells and come directly from the active muscle tissue [8]. The other factors are mainly mechanical and nutritional. Additionally, GI can significantly reduce the athlete’s capacity and performance, therefore developing new ways of supplementation especially focused on reducing these discomforts is of great importance. Dietary strategies may improve the physical comfort of athletes and reduce their risk of GI, e.g., probiotic supplementation is one potential strategy for reducing GI symptoms during endurance exercise [9].

There is some evidence that probiotics can be effective and safe for both preventing and treating GI complaints caused by exhaustive sports activity [10], as well as increase physical performance by maintaining gastrointestinal and immune function, thus reducing the susceptibility to illness [11,12]. The International Society of Sports Nutrition (ISSN) also put forward the position that certain probiotic strains may reduce the severity of GI disturbance when they occur [13]. However, initial research into the efficacy of probiotics in physically active populations has thus far been inconclusive. A few studies have indicated that probiotic supplementation might be useful for enhancing immunity and reducing the duration of GI illness in endurance-based athletes [14], but this has not been supported by other studies [15,16]. Indeed, even moderate-intensity exercise of short duration can compromise the GI tract and promote the occurrence of GI symptoms.

Given current knowledge, this study evaluated the effectiveness of probiotic supplementation on GI complaints, loss of barrier function, and cytokine levels in athletes using the data from randomised controlled trials. This systematic review could assist further research into the effects of probiotic supplementation on GI complaints in athletes and the development of dietary guidelines for these populations.

## 2. Methods

### 2.1. PRISMA Guidelines and the PICO Principle

This systematic review was designed following the Preferred Reporting Items for Systematic Reviews (PRISMA) recommendations and registered in the International Prospective Register of Systematic Reviews (PROSPERO) (number CRD42021284938). Inclusion criteria were based on the PICOS framework:

(1) studies involving healthy adult athletes of both sexes who did physical activity;
(2) interventions with probiotics, prebiotics, and synbiotics (the PRO group) with detailed information about the dose of supplementation, strain, and strain designation;
(3) inclusion of a control/placebo group (the PLA group);
(4) outcomes not previously defined (as an open question; all outcomes evaluated by the included studies were reported);
(5) randomised clinical trials (crossover or parallel), with no language or date restrictions.

Where data were incomplete, authors were contacted to obtain the relevant information. The PICOS criteria were defined as shown in Table 1.

### 2.2. Literature Sources, Search Strategy, and Selection Criteria

An electronic search of the literature was undertaken by SzK using three databases (PubMed, the *Cochrane Library*, and Web of Science) to identify all relevant articles. The search for articles was conducted between February 2019 and October 2021 using the terms “probiotics” OR “probiotic agent” OR “prebiotic” OR “synbiotic” OR “synbiotic agent” OR “beneficial microbes” OR “beneficial bacteria” OR “probiotic supplementation” OR “gut microbiota” AND “exercise training” OR “athletes” OR “sport” OR “exercise performance” AND “GI” OR “gastrointestinal complaints” OR “gastrointestinal symptoms” OR “gastro-intestinal complaints” OR “gastrointestinal symptoms”.

**Figure 1:**
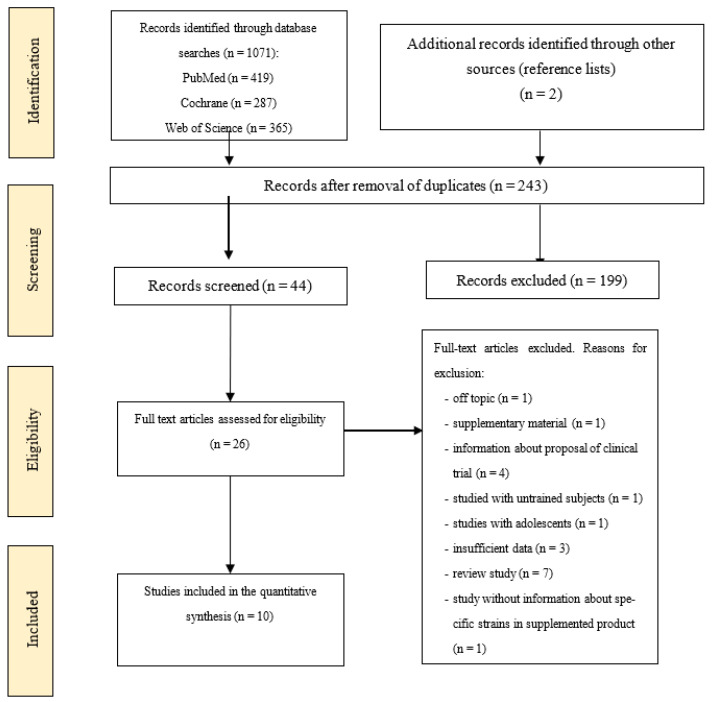
Flow diagram showing the study selection process.

Following the removal of duplicates, a two-phase search strategy was subsequently employed by two independent reviewers (KŁ and JB). In phase one, we assessed the eligibility of the studies based on the title and abstract of every hit generated from the search terms, comparing them against the inclusion and exclusion criteria. Studies that had questionable suitability were included with a final decision reached in phase 2. In phase 2, the full articles were retrieved and assessed against the eligibility criteria. Reference lists of original and review articles were screened to ensure that all relevant studies were included. Any differences of opinion relating to study eligibility were resolved through discussion. The search strategy is summarised in Figure 1.

### 2.3. Data Extraction and Quality Assessment

Two independent reviewers (KŁ, SzK) extracted the data and assessed the titles, abstracts, and full texts of the publications. Any disagreements that arose were solved through arbitration or consensus by a third reviewer (JB). For each study, the following data were collected: publication year, name of the first author, sample size, study design, a full description of the athletes (age, sex), the interventions used (including frequency, dose, strain, and strain designation of probiotic supplementation), the control interventions, and the main outcomes (GI symptoms, gut barrier, and inflammatory parameters). If the authors did not provide data on the specific strains, we contacted the producers of the supplements. Studies without information about dose and strain designation were excluded. Two authors (KŁ and JB) independently assessed the risk of bias in each study using the latest version of the Cochrane Collaboration Risk of Bias tool for RCTs, both for parallel-group trials and for the crossover trial template (RoB 2) [17] in line with the Cochrane Handbook’s criteria for judging bias risk [18]. Studies were assessed across the five domains for parallel-group and crossover trials: bias arising from the randomisation process; bias due to deviations from the intended interventions; bias due to missing outcome data; bias in measuring the outcome; and bias in selecting the reported result. The tool includes algorithms that map the responses to signalling questions onto a proposed risk of bias judgement for each domain across three levels: low risk of bias, some concerns, and high risk of bias. During the data abstraction process, attempts were made to contact the authors for further information beyond what had been published.

## 3. Results

### 3.1. Study Selection

A total of 1071 studies were identified using the PubMed, Cochrane, and Web of Science databases, and additional records (*n* = 2) were identified through the reference lists. A total of 1073 articles were excluded as duplicates or based on the titles or abstracts. Ultimately, 26 articles were screened for their full text, of which 18 full text articles were excluded for the reasons reported in Figure 1. By the end of the process, ten papers met the inclusion criteria and were included in the final analysis.

### 3.2. Population and Study Characteristics

A flow chart showing the study extraction process is presented in Figure 1. Most studies used supplements in the form of capsules [9,14,16,19,20,21,22,23] or beverages [15,24]. The number of study participants, the participant characteristics, the duration of the intervention, the type of supplement, and the sports discipline are presented in Table 2. A total of 822 study participants were included in the ten selected studies. The mean age of participants ranged from 23 (4) to 37 (11) years and they were mainly runners, cyclists, swimmers, triathletes, and rugby players. The interventions involved supplementation with probiotic bacteria such as *Bacillus subtilis* R0179 [21], *Bifidobacterium animalis* subsp. *lactis* CUL34 [14,20,22,23], CUL34, NCIMB 30,153 [9], Bi-04, Bi-07 [16], *Bifidobacterium animalis* ssp. *lactis lafti* B94 [21], *Bifidobacterium bifidum* CUL20, NCIMB 30,172 [9], CUL20 [14,20,22,23], *Bifidobacterium longum* R0175 [21], *Bifidobacterium breve* CUL69 [22], *Enterococcus faecium* R0026 [21], *Lactobacillus acidophilus* CUL60, CUL21 [14,20,22,23], NCFM [16], CUL60, NCIMB 30157, CUL21 NCIMB 30,156 [9], *Lactobacillus casei* CUL06 [20], CUL07 [22], *Lactobacillus casei Shirota (LcS)* [24], *Lactobacillus fermentum* VRI–003 PCC^®^ [19], CUL67 [20,22], *Lactobacillus helveticus Lafti* L10 [21], *Lactobacillus plantarum* CUL66 [20,22], *Lactobacillus rhamnosus* LGG, ATCC 53,103 [15], CUL63 [20], CUL66 [22], *Saccharomyces boulardi* (*S*. *cerevisiae*) [20], and *Streptococcus thermophilus* CUL68 [20,22]. One study also used fructooligosaccharides [9]. Eight studies were designed as parallel trials and two as crossover trials. Although there was no restriction on the date of publication, all articles included in this systematic review were published after 2007.

Most of the studies (80%) described the randomisation process in sufficient detail and were judged as having a low risk of bias in this domain^.^ [9,14,15,16,19,20,21,24] (see Supplementary Figures S1 and S2 for full details).

Due to the nature of the data, the limited number of studies, and the great heterogeneity of the studies which included various designs, population characteristics, and comparisons, it was decided to systematically summarise the current evidence and not to perform a quantitative meta-analysis (Table 3).

### 3.3. Number of GI Symptoms

Eight studies evaluated the number of GI symptoms [9,14,15,16,19,20,21,22], with six studies assessing the effects of probiotics on the number of GI symptoms during training [9,14,15,16,19,21]. In this case, no statistical difference in the number of GI symptoms was observed between the two studies [15,16].

Pugh et al. reported a lower prevalence of moderate GI symptoms during the third and fourth weeks of the probiotic supplement period than in the first and second weeks [23]. Roberts et al. also reported significantly lower overall symptom counts for training-related GI issues in the probiotic supplement group at the end of months 1 (*p* = 0.013) and 2 (*p* < 0.001) compared to the placebo group [9]. Likewise, Schreiber et al. found a significantly lower incidence of GI symptoms during training in the probiotic group than in the placebo group (*p* = 0.04) [21]. Furthermore, specific GI symptoms were compared separately, revealing significantly fewer incidences of nausea (*p* = 0.01), belching (*p* = 0.04), and vomiting (*p* = 0.04) in the probiotic group than in the placebo group [21].

In contrast, West et al. observed a two-fold increase in the number of mild (low-grade) self-reported GI symptoms in male and female non-elite cyclists and triathletes taking *L. fermentum* (VRI–003 PCC^®^). However, there was no substantial effect of supplementation evident between the groups with moderate and severe GI symptoms [19].

The effects of probiotics on GI during competitions and individual events were evaluated only in two studies [20,22], with one study assessing the effects of supplementation on the number of symptoms after the competition [15]. No significant differences were found between the probiotic and placebo groups.

### 3.4. The Proportion of Subjects with GI Symptoms (%)

Two studies evaluated the proportion of days during which subjects suffered from GI symptoms during training or (in one study) after the competition with no difference observed between the probiotic and placebo groups [15,24].

### 3.5. Total Symptom Severity of GI

Five studies evaluated the total symptom severity score of GI symptoms [9,14,19,23,24], of which three studies evaluated the impact of probiotics on the total GI symptom severity score during training [9,19,24]. In the study by Roberts et al., the average symptom severity was significantly lower in the probiotic group after months 1 and 3 of intervention than in the placebo group (*p* < 0.001) [9]. Furthermore, West et al. showed the self-reported severity score for GI illnesses at mean training load in males on probiotics to be 0.7 of a scale step lower than for males on the placebo, and this positive effect of the probiotic increased with the training load [19]. Pugh et al. and Schreiber et al. found no significant differences in total symptom severity score of GI during [23] and after competition [21]. Pugh et al. observed a lower total GI symptom severity score in the probiotic group during the final third of the race could have increased the average relative speed in the probiotic group compared to the placebo group [14].

### 3.6. Duration of GI Symptoms

Three studies evaluated total GI symptom severity [15,19,24]. Gleeson et al. [24] and Kekkonen et al. [15] found no significant difference in symptom duration between the probiotic and placebo groups. However, West et al. [19] reported a two-fold increase in the duration of mild (low-grade) self-reported gastrointestinal symptoms for male and female subjects taking *L. fermentum* (VRI–003 PCC^®^). Although Kekkonen et al. [15] found significant differences in the duration of the gastrointestinal symptom two weeks after running a marathon, the duration of these GI episodes was 57% shorter in the probiotic group compared to the placebo group (1.0 vs. 2, 3 d; *p* = 0.046).

### 3.7. Influence of Probiotic Supplementation on Inflammatory Markers and Gut Barrier Function

Six studies evaluated the effects of probiotic supplementation on inflammatory markers [14,19,21,22,23], with Pugh et al. [23], Schreiber et al. [21], and Shing et al. [22] reporting no significant differences in TNF-α or IL-6. Pugh et al. [14] and West et al. [19] observed significant decreases in the inflammatory parameters. Interestingly, supplementation with *Lactobacillus casei* Shirota decreased the TNF-α or IL-6 levels in the probiotic group after eight weeks of intervention, but after sixteen weeks, there were no significant differences between the probiotic and placebo groups. Furthermore, four studies [9,14,22,23] evaluated changes in gut barrier function after probiotic supplementation, but no statistical differences were observed between the study groups.

## 4. Discussion

Endurance athletes exposed to high-intensity exercise often experience GI symptoms frequently associated with alterations in intestinal permeability, decreased gut barrier function, and systemic responses (i.e., endotoxemia and cytokinemia) [3,25]. This may occur because exercise affects the gut transit time and intestinal immune response, which could modify the microbiota composition and metabolic activity [25]. Although such symptoms are generally not severe enough to prevent athletes from exercising, they may cause athletes to reduce the intensity of exercise, which in turn can impair exercise performance and ultimately their sports results [5].

The present systematic review summarised the available RCTs on the effects of probiotics (single or multiple strains) on GI problems during training, competition, and single event, and immediately after in different groups of athletes, but mainly runners, cyclists, swimmers, triathletes, and rugby players. Overall, ten RCTs published between 2007 and 2021 fulfilling our criteria were included in this review.

During training, the number of GI symptoms was significantly reduced compared to a matched placebo in three out of seven studies where probiotic supplementation was provided. In turn, the severity of GI symptoms was considerably reduced in two out of three studies. In none of the studies was the proportion of participants who took probiotics and experienced GI symptoms lower than among those who took placebos, and in none of the two studies was the duration of GI symptoms shorter with probiotic supplementation. Moreover, West et al. observed that the frequency and duration of mild (low-grade) self-reported GI symptoms during eleven weeks of training were twice as high in the probiotic group as in the placebo group [19]. The increased frequency of GI symptoms may reflect the short-term adaptive responses in the GI tract with probiotic use, especially among women [19]. Otherwise, improvements in the number and severity of GI symptoms during training were observed in studies involving runners, cyclists, and triathletes [9,14,19,21]. In these studies, probiotic supplementation was performed with the use of both single strain and multi-strain probiotics, *Bacillus subtilis* R0179 [21], *Bifidobacterium animalis subsp. lactis* CUL34, NCIMB 30,153 [9], CUL34 [14], *Bifidobacterium animalis ssp. lactis lafti* B94 [21], *Bifidobacterium bifidum* CUL20, NCIMB 30,172 [9], CUL20 [14], *Bifidobacterium longum* R0175 [21], *Enterococcus faecium* R0026 [21], *Lactobacillus acidophilus* CUL60, CUL21 [14], CUL21 NCIMB 30,156 [9], *Lactobacillus fermentum* VRI–003 PCC^®^ [19], and *Lactobacillus helveticus Lafti* L10 [21] with a duration of 13 to 16 weeks [9,14,19,21].

Nonetheless, the findings collected in our review indicate that probiotic bacteria may have only a moderate impact on reducing both the occurrence and severity of GI symptoms during training, which is opposed to earlier findings [26]. In the case of competition and test exercise, none of the studies showed the capacity of probiotic bacteria supplementation to reduce the incidence of GI symptoms. In only one of the three studies did supplementation for four weeks with a *Lactobacillus* and *Bifidobacterium* multi-strain cocktail (*Lactobacillus acidophilus* CUL60, *L. acidophilus* CUL21, *Bifidobacterium bifidum* CUL20, and *Bifidobacterium animalis subsp. Lactis* CUL34) reduce by a third the severity of GI symptoms compared to the placebo during the final third of a marathon, indicating that such supplementation strategies may help maintain running pace during the latter stages of racing [14].

After the competition, GI symptom occurrence in the probiotic group was significantly reduced over the placebo in none of the studies. In the study of Kekkonen et al. [15], there were also no differences between the groups in terms of the number of GI episodes during the two weeks after the marathon. During the same period, however, the duration of a GI-symptom episode was 57% shorter in the *Lactobacillus rhamnosus* GG ATCC 53,103 group than in the placebo group (1.0 vs. 2.3 d; *p* = 0.046).

Intense exercise may increase the permeability of the gut, allowing the translocation of toxins from the gut into the systemic circulation. One consequence of this gut permeability is increased levels of bacterial endotoxins, which coincide with increases in inflammatory cytokines. Indeed, it has been reported that runners with the highest post-race endotoxin levels were four times more likely to experience GI symptoms than runners with the lowest endotoxin levels [27]. It has thus been suggested that improving barrier function through probiotic supplementation should enhance resilience against exercise-induced endotoxemia and inflammation, and possibly also against gastrointestinal problems. However, none of the studies found any additional positive impact of probiotic supplementation on gut barrier function. It should however be noted that in the study of Roberts et al., the multi-strain probiotic supplementation was also accompanied by the use of prebiotics that has been shown to enhance probiotic efficacy by maintaining intestinal permeability (assessment from urinary lactulose:mannitol (L:M) ratio measurement) [9]. Coman et al. observed that supplementation with a mixture containing two probiotics (*L. rhamnosus* IMC 501 and *L. paracasei* IMC 502) and a prebiotic (oat bran fibre) was associated with an increase in *Lactobacilli* and *Bifidobacteria*, which in turn may have improved intestinal permeability in the participants [28]. Whereas recreational and moderate exercise can have anti-inflammatory and immunomodulatory effects, the intense exercise of elite athletes can induce inflammation through the synthesis and release of many cytokines (IL-6, IL-1ß, macrophage inflammatory protein-1a, IL-8, TNF-α, IL-10, and IL-1 receptor antagonist) [29]. In our earlier meta-analysis of the effectiveness of probiotic supplementation on the upper respiratory tract infection (URTI) and inflammatory markers in elite athletes, we concluded that probiotic supplementation may decrease IL-6 and TNF- levels [30]. The beneficial effects of probiotic supplementation on IL-6 and TNF-α levels are particularly important for athletes, as an increase in IL-6 secretions causes inflammation, fatigue, pain, mood changes, and concentration disorders which may worsen performance [13,31,32]. The post-exercise increases in plasma TNF-α also suggest a source in damaged muscles [33]. Only two of six studies in this systematic review showed significant decreases in inflammatory parameters [19,23]. Additionally, Gleeson et al. found that probiotic supplementation decreased TNF-α or IL-6 levels after eight weeks of intervention, but by sixteen weeks, there were no differences between the probiotic and placebo groups [24].

The review by Miles [26] indicates that probiotic supplementation can promote improvements in sports performance through various pathways in athletes and physically active individuals using discrete strains of probiotics. In our systematic review, most studies did not confirm probiotic supplementation to have positive effects on sports performance, nor did we observe any association with GI symptoms. In the study of Pugh et al., where multi-strain probiotic supplementation (*Lactobacillus acidophilus* CUL60, *Lactobacillus acidophilus* CUL21, *Bifidobacterium bifidum* CUL20 and *Bifidobacterium animalis subsp. Lactis* CUL34) was taken for 28 days before a marathon, the lower total GI symptom severity score in the probiotic group during the final third of the race could have increased the average relative speed compared to the placebo group [14].

The strength of our systematic review is that studies without detailed information about strain designation were excluded to allow a choice of an appropriate type of probiotic by athletes, coaches, and medical care professionals. Nonetheless, this study had some limitations. First, the number of eligible studies was small, and most trials were performed in a small sample size population, with a short duration of supplementation, therefore the results should be considered with caution. Second, using many diverse supplementation protocols in terms of doses, strains, forms (capsules, sachets, or drinks), methods to identify specific GI symptoms and the lack of adequate dietary controls make it difficult to interpret the clinical effects of probiotic supplementation on GI symptoms, the gut barrier function, and inflammatory markers in athletes. Therefore, transparent and rigorous supplementation protocols and measurement methodologies are needed when evaluating the effects of probiotic supplementation on the gut health of athletes.

## 5. Conclusions

This systematic review found little evidence for probiotic supplementation improving GI symptoms in athletes during exercise training and competition. Although different probiotics were tested, the current evidence does not allow the identification of the best product for managing GI symptoms in athletes. Furthermore, due to the small number of studies available, it is difficult to find a direct association between reduced exercise-induced perturbations in cytokines and gut barrier function, and GI symptoms after probiotic supplementation. Thus, there is a need for well-designed human intervention studies to clarify the impact of probiotic supplementation on gut-related issues among athletes. These studies should also evaluate markers related to gut barrier function and inflammation. In addition, it would be interesting to understand how probiotics alone, or in combination with prebiotics, may affect the exercise performance of athletes.

## Figures and Tables

**Table 1:** PICOS criteria for inclusion of studies.

PICOS Criteria	Definition of Criteria for Studies
Participants	Athletes (aged ≥ 18 years)
Intervention	Oral supplementation with probiotics, prebiotics, symbiotics
Comparator	Control/placebo
Outcomes	Primary outcome: gastrointestinal symptoms Secondary outcomes: IL-6, TNF-α, gastrointestinal permeability,
Study design	RCT randomised controlled trial

**Table 2:** Characteristics of studies and populations.

Study	Design	Population (*n*), Age	Supplementation Protocol			Control/ Placebo	Duration	Limitation of the Study
Gleeson et al. (2011) [24]	P	Runners, cyclists, swimmers, triathlons, racket sports, team gamers (male and females) PRO: *n* = 32; 32 (14) PLA: *n* = 26; 2 (9) All: 27.0 (11.6)	*Lactobacillus casei Shirota* (LcS) (6.5 × 10^9^ CFU) per probiotic drink	2 probiotic drinks per day	single strain	drink without *Lactobacillus casei Shirota*	16 weeks	
Kekkonen et al. (2007) [15]	P	Marathon runners (male & female) PRO: *n* = 61; 40 (22–58) PLA: *n* = 58; 40 (23–69)	*Lactobacillus rhamnosus* LGG, ATCC 53,103 (3 × 10^8^ CFU) in milk-based fruit drink or *Lactobacillus rhamnosus* LGG, ATCC 53,103 (5 × 10^9^ CFU) per capsule	2 milk-based fruit drinks per day (4 × 10^10^ CFU) or 2 capsules per day (1 × 10^10^ CFU)	single strain	milk-based fruit drink or capsule without probiotic bacteria	12 weeks	
Pugh et al. (2019) [14]	P	Runners (male & female) PRO: *n* = 12; 34.8 (6.9) PLA: *n* = 12; 36.1 (7.5)	*Lactobacillus acidophilus* CUL60, *L. acidophilus* CUL21, *Bifidobacterium bifidum* CUL20, & *Bifidobacterium animalis subsp. Lactis* CUL34 (2.5 × 10^10^ CFU) per capsule	1 capsule per day	multi-strain	cornstarch	4 weeks	lack information about sweat rates and levels of dehydration and core temperature which can affect GI symptoms
Pugh et al. (2020) [23]	C	Cyclists (male) PRO: 7 PLA: 7 All: 23 (4)	*Lactobacillus acidophilus* CUL60, *L. acidophilus* CUL21, *Bifidobacterium bifidum* CUL20, & *Bifidobacterium animalis* subsp. *lactis* CUL34 (2.5 × 10^10^ CFU) per capsule	1 capsule per day	multi-strain	cornstarch	28 days	small sample size and lack of statistical power
Pumpa et al. (2019) [20]	P	Elite rugby union athletes PRO: *n* = 9; 27.0 (3.2) PLA: *n* = 10; 26.6 (2.9)	*Lactobacillus rhamnosus* CUL63 (1.555 × 10^10^ CFU), *Lactobacillus casei* CUL06 (9.45 × 10^9^ CFU), *Lactobacillus acidophilus* CUL21 + CUL60 (2 × 10^10^ CFU), *Lactobacillus plantarum* CUL66 (3.15 × 10^9^ CFU), *Lactobacillus fermentum* CUL67 (1.35 × 10^9^ CFU), *Bifidobacterium* *animalis* subsp. *lactis* CUL 34 (6.55 × 10^9^ CFU), *Bifidobacterium bifidum* CUL20 (3.45 × 10^8^ CFU)*, Streptococcus thermophilus* CUL68 (2.25 × 10^9^ CFU) and *Saccharomyces boulardi (S. cerevisiae)* (250 mg) per capsule	1 capsule per day	multi-strain	microcrystalline, iron oxide yellow, iron oxide red, gelatin capsule; and SB Floractiv: microcrystalline cellulose, lactose, calcium hydrogen phosphate dihydrate, povodine, silica colloidal anhydrous, magnesium stearate, gelatin capsule	17 weeks	
Roberts (2016) [9]	P	Male and female triatheletes PRO: *n* = 10; 35 (2) PLA: *n* =10, 35 (3)	*Lactobacillus acidophilus* CUL60, NCIMB 30157 (1 × 1010 CFU) *Lactobacillus acidophillus* CUL21, NCIMB 30156 (1 × 1010 CFU), *Bifidobacterium bifidum* CUL20, NCIMB 30172 (9.5 × 10^9^ CFU) *Bifidobacterium animalis subsp. lactis* CUL34, NCIMB 30153 (5 × 10^8^ CFU) and fructooligosaccharides (55.8 mg) per capsule	1 capsule per day	multi-strain + prebiotic	200 mg cornflour	12 weeks before and 6 days after a triathlon	-
Schreiber et al. (2021) [21]	P	Male cyclists PLA: *n* = 11 PRO: *n* = 16 (19-40)	*Lactobacillus helveticus Lafti* L10 (4.3 × 10^9^ CFU), *Bifidobacterium animalis subsp. lactis Lafti* B94 (4.3 × 10^9^ CFU), *Enterococcus faecium* R0026 (3.9 × 10^9^ CFU), *Bifidobacterium longum* R0175 (2.1 × 10^9^ CFU) & *Bacillus subtilis* R0179 (0.4 × 10^9^ CFU) per capsule	1 capsule per day	multi-strain	capsules contained the excipients only (potato starch, magnesium stearate, ascorbic acid, and white vegetable powder) without the bacteria	90 days/~13 weeks	small sample size the cyclists were at various phases of their training/completion season thus, some were at their peak competition level while others were training for their upcoming competitions season
Shing et al. (2014) [22]	C	Male runners PRO: *n* = 5 PLA: *n* = 5 All: 27 (2)	*Lactobacillus acidophilus* CUL21 + CUL60 (7.45 × 10^9^ CFU), *Lactobacillus rhamnosus* CUL66 (1.555 × 10^10^ CFU), *Lactobacillus casei* CUL07 (9.45 × 10^9^ CFU), *Lactobacillus plantarum* CUL66 (3.15 × 10^9^ CFU), *Lactobacillus fermentum* CUL67 (1.35 × 10^9^ CFU), *Bifidobacterium animalis subsp. lactis* CUL34 (4.05 × 10^9^ CFU), *Bifidobacterium breve* CUL69 (1.35 × 10^9^ CFU), *Bifidobacterium bifidum* CUL20 (4.5 × 10^8^ CFU), & *Streptococcus thermophilus* CUL68 (2.25 × 10^9^ CFU) per capsule	1 capsule per day	multi-strain	skim milk powder	4 weeks	small sample size and only included males, short study duration of 4 weeks
West et al. [19]	P	Male and female cyclists and triathletes (not elite) PRO: *n* = 29; 35.2 (10.3) PLA: *n* = 33; 36.4 (8.9) 35 (9)	*Lactobacillus fermentum* VRI–003 PCC^®^ (1 × 10^9^ CFU) per capsule	1 capsule per day	single strain	Microcrystalline cellulose	11 weeks	
West et al. [16]	P	Athletes (male and female) PRO: *n* = 161; 36 (12) PRO1: *n* = 155; 36 (11) PLA: *n*= 149; 37 (11)	PRO: *Bifidobacterium* *animalis subsp. lactis* Bi-04 (2.0 × 10^9^ CFU) per sachet PRO1: *Lactobacillus acidophilus* NCFM (5.0 × 10^9^ CFU) and *Bifidobacterium animalis subsp*. *lactis* Bi-07 (5.0 × 10^9^ CFU) per sachet	PRO: 1 sachet per day PRO1: 1 sachet per day	PRO: single strain PRO1: multi-strain	sucrose base without the probiotic bacteria	11 weeks	

PRO: probiotic group. PLA: placebo group. P: parallel. C: crossover.

**Table 3:** Impact of probiotics on GI symptoms, inflammatory markers, and gut barrier function during training, competition, or single event and after the competition.

Authors	Number of GI Symptoms	The Proportion of Subjects with GI Symptoms (%)	Total Symptom Severity Score of GI	Duration of Symptoms (Days)	Gut Barrier Function	TNF-α	IL-6
Impact of probiotics on GI symptoms during training							
Gleeson et al. 2011 [24]	-	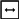	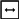	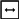	-	↓ (after 8 weeks of intervention) 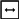 (after 16 weeks of intervention)	↓ (after 8 weeks of intervention) 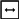 (after 16 weeks of intervention)
Kekkonen et al. 2007 [15]	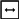	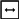	-	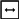	-	-	-
Pugh et al. 2019 [14]	↓	-	-	-	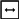	-	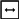
Roberts et al. 2016 [9]	↓	-	↓	-	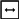	-	-
Schreiber et al. 2021 [21]	↓	-	-	-	-	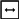	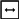
West et al. 2011 [19]	↑	-	↓	↑	-	↓	↓
West et al. 2014 [16]	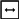	-	-	-	-	-	-
	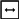	-	-	-	-	-	-
Impact of probiotics on GI symptoms during competition or single event							
Kekkonen et al. 2007 [15]	-	-	-	-	-	-	-
Pugh et al. 2019 [14]	-	-	↓ (during final 1/3 of marathon race)	-	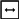	-	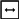
Pugh et al. 2020 [23]	-	-	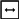	-	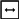	-	↓
Pumpa et al. 2019 [20]	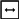	-	-	-	-	-	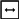
Roberts et al. 2016 [9]	-	-	-	-	-	-	-
Schreiber et al. 2021 [21]	-	-	-	-	-	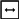	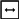
Shing et al. 2014 [22]	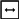	-	-	-	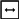	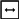	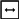
Impact of probiotics on GI symptoms after the competition							
Kekkonen et al. 2007 [15]	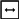	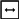	-	↓	-	-	-
Pugh et al. 2019 [14]	-	-	-	-	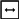	-	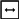
Schreiber et al. 2021 [21]	-	-	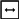	-	-	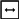	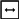

↓ a significant decrease in effect. 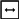 there was no (significant) effect. ↑ a significant increase in effect.

## Data Availability

All data supporting the reported results can be found in this publication and the Supplementary Materials.

## Supplementary Materials

The following supporting information can be downloaded at: https://www.mdpi.com/article/10.3390/nu14132645/s1, Figure S1: Risk of bias and applicability concerns parallel studies; Figure S2: Risk of bias and applicability concerns crossover studies.
