# Supplementary material for: Effects of Probiotics Supplementation on Gastrointestinal Symptoms in Athletes: A Systematic Review of Randomized Controlled Trials
Source: Nutrients. 2022 Jun 26;14(13):2645. doi: 10.3390/nu14132645 (PMC9268154; doi:10.3390/nu14132645)
Supplement: Supplementary file 1 [file nutrients-14-02645-s001.zip › nutrients-1775937-supplementary.pdf]

Supplementary materials

Figure S1. Risk of bias and applicability concerns parallel studies

**Unique ID**	**Study ID**	**D1a**	**D1b**	**D2**	**D3**	**D4**	**D5**	**Overall**
1	Gleeson et al. 2011	!	+	+	+	+	!	!
2	Kekkonen et al. 2007	+	+	!	+	+	+	!
3	Pugh et al. 2019	+	+	+	+	+	+	+
4	Pumpa et al. 2019	+	+	+	+	+	+	+
5	Roberts et al. 2016	+	+	+	+	+	+	+
6	Schreiber et al. 2021	+	+	+	+	+	+	+
7	West et al. 2011	+	+	+	+	+	+	+
8	West et al. 2014	+	+	+	+	+	+	+

+ Low risk

! Some concerns

High risk

Legend:

D1a	Randomisation process
D1b	Timing of identification or recruitment of participants
D2	Deviations from the intended interventions
D3	Missing outcome data
D4	Measurement of the outcome
D5	Selection of the reported result

Figure S2. Risk of bias and applicability concerns crossover studies

Unique ID	Study ID	D1	DS	D2	D3	D4	D5	Overall
1	Pugh et al. 2020	!	+	+	+	+	!	!
2	Shing et al. 2014	+	+	!	+	+	!	!

+ Low risk

! Some concerns

High risk

D1	Randomisation process
DS	Bias arising from period and carryover effects
D2	Deviations from the intended interventions
D3	Missing outcome data
D4	Measurement of the outcome
D5	Selection of the reported result
